# Hyperspectral-Enhanced Dark Field Microscopy for Single and Collective Nanoparticle Characterization in Biological Environments

**DOI:** 10.3390/ma11020243

**Published:** 2018-02-06

**Authors:** Paula Zamora-Perez, Dionysia Tsoutsi, Ruixue Xu, Pilar Rivera_Gil

**Affiliations:** Integrative Biomedical Materials and Nanomedicine Lab, Department of Experimental and Health Sciences (DCEXS), Pompeu Fabra University (UPF), PRBB, Barcelona 08003, Spain; paula.zamora@upf.edu (P.Z.-P.); ruixue.xu01@estudiant.upf.edu (R.X.)

**Keywords:** single-particle tracking, hyperspectral analysis of nanoparticles evolution, protein corona, colloidal stability, living organisms, scattering, correlating physicochemical properties with biological responses, enhanced dark field imaging

## Abstract

We review how the hyperspectral dark field analysis gives us quantitative insights into the manner that different nanoscale materials interact with their environment and how this relationship is directly expressed in an optical readout. We engage classification tools to identify dominant spectral signatures within a scene or to qualitatively characterize nanoparticles individually or in populations based on their composition and morphology. Moreover, we follow up the morphological evolution of nanoparticles over time and in different biological environments to better understand and establish a link between the observed nanoparticles’ changes and cellular behaviors.

## 1. Hyperspectral-Enhanced Dark Field Microscopy (HEDFM)

### 1.1. Dark Field Microscopy and Hyperspectral Imaging—A Brief Introduction 

Dark field microscopy anchored with hyperspectral imagery (HSI) is a novel optical approach with great potential in bio-related disciplines because it allows the identification and quantitative determination of specific components in the biological milieu. The optical approaches are universally employed methods as they offer a direct and reliable observation, however, they are limited by the resolution of the optical system [1]. Bright field optics rely on the transmitted light illumination, which is ideal for objects that absorb light against a non-absorbing background. This optical modality contemplates the reflected and transmitted light leading to a low optical resolution, decreased signal-to-noise ratios, and out-of-focus appearance of the sample. On the contrary, dark field microscopy contemplates only the incoming reflected light from the sample, thus eliminating the background noise [2]. Dark field microscopy provides a high contrast suitable for the observation of low-contrast objects, usually not visible by conventional bright field microscopy. It is based on the indirect illumination of the specimen and, upon interaction with the sample, collects only the reflected or elastically scattered light [3]. A specially sized disc blocks the illumination so that only oblique rays strike the sample, permitting the distinguishable visualization of objects with similar refractive indexes as the background. The latter characteristic is of interest especially for the microscopic examination of biological samples without contrast agents [4]. 

HSI is a powerful multitool expanding into the realm of biological and medical sciences, which holds a proven record of success in astronomy, geosciences, agriculture, surveillance, resource management, and environmental monitoring, among other applications. HSI yields a hyperdata cube with continuous spectral and spatial information in one measurement, engaging non-contact and remote sensing. Moreover, this method is sensitive to subtle spectral changes ensuring a thorough discrimination of chemical or biological entities, or tracking agents' changes over time [5]. The combination of the spatial-scanning hyperspectral imaging methodology with dark field microscopy is considered highly advantageous for optical studies of nanoscale materials. Although both HSI systems and dark field microscopy have been widespread methods for years, it was only recently that the combination of these two methods emerged in one single modality.

### 1.2. Instrumentation and Advantages

**Figure 1:**
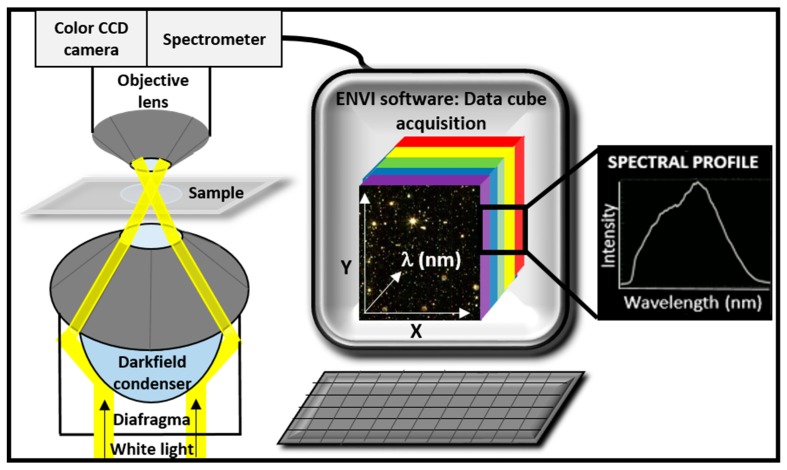
Generalized diagram of the Hyperspectral-Enhanced Dark Field Microscopy (HEDFM) system. Schematic illustration of the experimental setup based on dark field optics coupled with the spectrograph (**left**), and on the acquisition of the hyperspectral data cube for the analysis (**right**). Each pixel in the field of view provides complete spectral information in the optical window ranging from 400 to 1000 nm [8]. This system was acquired from CytoViva, Inc. (Auburn, AL, USA).

HEDFM has a powerful resolving capability and is designed to provide in situ optical observation and spectral characterization of a wide range of nanomaterials as they interact with both biological and material-based matrixes, a key aspect for many bioanalytical applications. The unique interaction between light and matter generates characteristic profiles of scattered light, serving as spectral signatures for each target. Dark field illumination based on an annular cardioid condenser and highly collimated light at oblique angles on the sample, generates images with enhanced contrast and signal-to-noise ratio up to ten times (×10) higher than the conventional dark field optics [6,7]. The instrumentation of the system consists of a visible-near infrared (vis-NIR) spectrophotometer attached to a charge-coupled device (CCD) camera connected with the imaging port of the microscope. Figure 1 provides a generalized scheme of the system and the data acquisition device [8]. This technology uses illumination from a tungsten–halogen light source capable to operate spectrally from 400 nm ≤ λ ≤ 1000 nm, Δλ < 4.69 nm, and with a diffraction-limited spatial resolution reaching a pixel size of a few nanometers for the 100X objective [9]. The image collection along with the analysis software provides evidence of the existence of nanoparticles (NPs) through the collection of all vis–near-infrared (NIR) spectral data within each pixel of the scanned area, storing the unique spectral signatures of the selected elements and forming a library database. Finally, the spectral signatures in the library database can be tracked against newly scanned samples, thus permitting high-accuracy identification of the element.

HEDFM allows real-time analysis and can be successfully implemented in various biomedical areas, for instance, bionanomaterials synthesis, nanodrug delivery, and nanotoxicology [10,11]. The main advantages include the nondestructive nature of the method and the direct sample testing with no need for processing steps prior to the analysis. A sensitive analysis is also possible because HEDFM enables long-term studies in live cells and tissues even under flow conditions mimicking the blood circulation.

### 1.3. Image Acquisition and Hypercube Analysis

The hyperspectral data cube acquisition provides three-dimensional data with two spatial (x, y) and one spectral (z) dimensions. The hyperspectral image thereby is considered the dark field image along with the spectral information associated with each pixel of the image. Different strategies may be employed for the data cube acquisition, depending on the aimed resolution of the method. In general, the commonly utilized methods in HSI from higher to lower resolution are: point mapping or “whiskbroom”, line scanning or “pushbroom”, plane scanning or “staredown”, and the non-scanning or “snapshot” method [5]. The image acquisition and data cube preprocessing for analysis involve different phases until the formation of a spectral library of endmembers, regardless of the engaged method [1,9]. Typically, the vis-NIR HSI systems engage mapping in a pushbroom fashion. The hypercube is generated in time intervals of seconds or minutes, upon adjusting the exposure time. 

**Figure 2:**
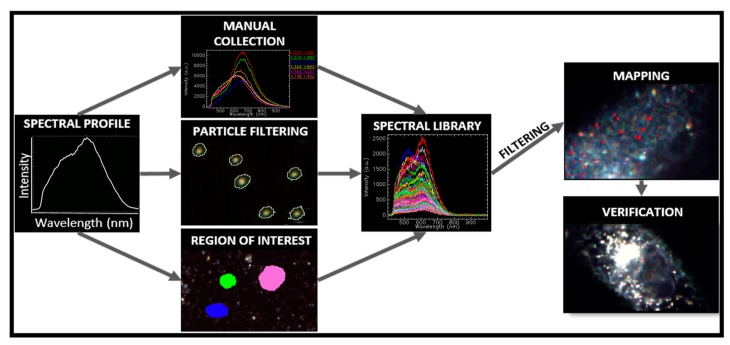
Workflow description of the data analysis and mapping. The spectral library can be generated either manually by individual selection of the acquired spectral profiles or through automated processes using software tools. The endmembers of the spectral library are tracked into unknown data cubes. This allows identifying the nanomaterials within biological matrixes. As a negative control for identification, cells unexposed to nanoparticles are mapped with the same spectral library [8].

Normally, a hypercube may contain hundreds of thousands of pixels with the size of a few nanometers each. A raw hypercube includes unwanted information, nonresponsive pixels, and spikes because of cosmic radiation that need to be carefully removed. Next, thresholding techniques are employed to visualize the image background at a wavelength that ensures enough contrast between the target and the background. Figure 2 summarizes the processes of the spectral endmember selection in the field of view [8]. Manual procedures allow the visualization of the spectral variability within a sample by comparing single spectra. The spectral selection using a particle-filtering tool either accomplishes the mean spectral profile of a targeted population or generates spectral-like sub-populations classified by pixel size. 

The region-of-interest (ROI) tool converts the selected pixels into a spectral library or endmember collection file for the subsequent mapping analysis. The peak location classifier detects the pixels in a specific wavelength range allowing quantitative examination. For example, the size characterization of spherical gold nanoparticles (AuNPs) is possible across a wide optical window. The image pixels are analyzed in a desired wavelength range and are further classified upon their spectral responses. Colors can be associated to each range for the ease of identification of spectrally different nanoparticle subpopulations or spectral changes in individual nanoparticles. The Spectral Angle Mapper (SAM) is an automated mapping method acting as a threshold of spectral angle. The algorithm determines the spectral similarity and provides information about the location and analogy of endmember pixels in an input image. The spectral angle between the endmember and the image pixels defines the degree of similarity between known and unknown pixels. The SAM classifier is a powerful exploratory tool enabling the investigation of a new scene. 

In this review, we introduce the reader to how HEDFM can track interfacial changes at the level of individual and collective nanoparticles. Factors affecting the interface dispersant (NP)-dispersion medium are, for example, the composition of the medium, NPs’ stabilization procedure, NPs’ surface chemistry. This technique provides a tool to correlate changes in the physicochemical properties of colloidal nanoparticles caused by environmental (ionic strength, viscosity, pH, lyophility, cells) and non-environmental factors, such as the time, upon the identification of the corresponding spectral signatures. 

#### 1.3.1. Optical Response of Multicomposite Nanoparticles 

**Figure 3:**
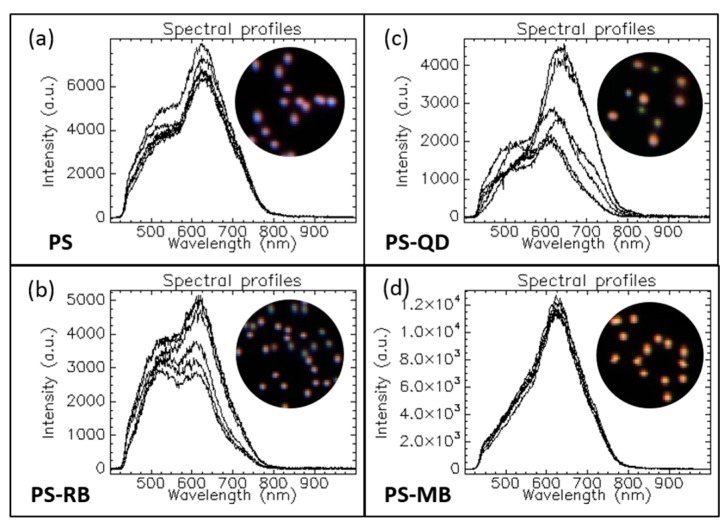
Six spectral profiles showing optical differences among randomly selected PS NPs encapsulating different cargos. The insets correspond to dark field images of the NPs. (**a**) Bare PS NPs; (**b**) PS loaded with rhodamine (RB); (**c**) PS loaded with quantum dots (QD); (**d**) PS loaded with methylene blue (MB). The results were obtained using a CytoViva^®^ with an Olympus UPlan FLN 60X 1.25 N.A. oil objective, a tungsten–halogen lamp as the illumination source, and a Pixelfly hyperspectral camera based on pushbroom line scanning.

High speed and reliable screening, localization, and chemical identification of various components is the key in nanomedicine and biotechnology. Morphology and composition define the scattering properties of each compound which are then associated to a characteristic spectral signature. Figure 3 presents an example of HEDFM enabling the spectral differentiation of polystyrene nanoparticles (PS NPs) through the encapsulation of different cargoes (samples a–d). Bare PS NPs (to a lesser extent) (Figure 3a) and PS NPs loaded with rhodamine (RB) (Figure 3b) or quantum dots (QD) (Figure 3c) exhibited spectral variability among six individual NPs. This could be seen in the different spectral profiles associated to each NP, whereas PS loaded with methylene blue (MB) (Figure 3d) did not show interparticle variability. The insets are red-green-blue (RGB) images of the NPs according to their spectral profiles to highlight the presence of spectrally different subpopulations. The mean spectral profile of each sample is shown in Figure S1 (see supporting information). Variations in the composition or geometry can be reflected directly in the spectral readout of individual or collective NPs. From the mean spectral profiles of each sample, we could analyze the impact of the loading on NPs’ behavior (Figure S1). First, the reference spectra collected from the unloaded NPs showed a primary band around 600–630 nm, which was maintained across the samples (Figure 3a–d) regardless of the loading (Figure S1). When the NPs were loaded with the different cargoes, their scattering spectra, compared to the spectra of unloaded NPs, did not show a shift, indicating sample homogeneity and no interparticle variability. The subtle blue shifting (Figure S1) of the PS–RB could be associated to a slight redispersion of the individual NPs. The appearance of the shoulder in the 500–550 nm region was associated with the loading. The variability between the spectra of the six PS–RB NPs indicated that the amount of RB loaded was not the same for each NP, permitting the spectral classification of PS NPs with a higher RB cargo. PS–QD spectral analysis did not show a specific trend (Figure 3c), indicating a different number of QDs per PS bead. QD loading produced enough changes on the physicochemical properties of the NPs to induce variations in the scattering profiles. This seems feasible considering that RB and QDs are between 0.05 nm and 40 nm, respectively. This seems to be a random process associated to an inhomogeneous distribution or loading of the QD within the PS NPs. The highest impact on the spectral profile of the PS NPs was registered upon MB loading. The spectral shoulder completely disappeared, maintaining the main spectral band at 620 nm (Figure 3d). Moreover, reproducible spectral profiles were collected for the PS–MB NPs, indicating homogeneous MB cargo per PS NP. These results confirmed that HEDFM can spectrally track the physicochemical changes induced in nanoparticles.

#### 1.3.2. Tracking NPs’ Evolution at Different pH Values

**Figure 4:**
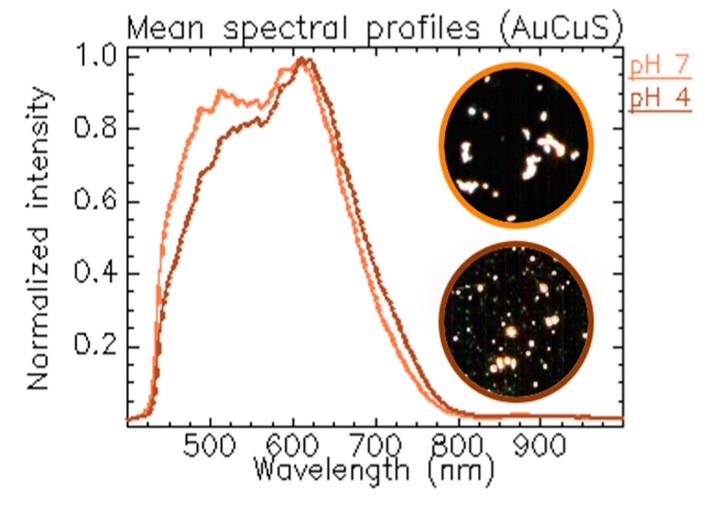
Spectral responses of AuCuS NPs at different pH values [8]. Mean spectral profiles of the particles in neutral (orange line) and acidic (brown line) pH conditions. The insets correspond to the dark field images of the AuCuS NPs. CytoViva setup and ENVI software (Version 4.8) were used for the hypercube acquisition and analysis.

The surface of engineered nanoparticles synthesized using bottom-up protocols is normally modified (e.g., coated with polymers) to provide colloidal stability in water-based media and to further functionalize the NP’s surface. The surface chemistry governs the interaction of the NPs with the environment (medium, cells, and other), therefore its nature defines the NPs’ optical response. In aqueous conditions, many protective coatings present sensitive functional groups for the electrostatic stabilization of the particles that are highly affected by pH. We engaged a detailed HEDFM analysis to examine these differences using gold-copper sulfide nanoparticles (AuCuS NPs) (Figure 4 and Figure S2) [8]. Our results showed a narrowing and a subtle red shifting of the two main bands of the spectral signature of the AuCuS NPs at acidic pH compared to neutral pH. We estimated that the observed spectral changes were related to the protonation state of the protective polymer. The swelling–shrinking behavior of polymers affects the local refractive index, thereby modifying the optical responses of the nanomaterials.

#### 1.3.3. Tracking NPs’ Changes Induced by the Solvent’s Ionic Strength and Composition

**Figure 5:**
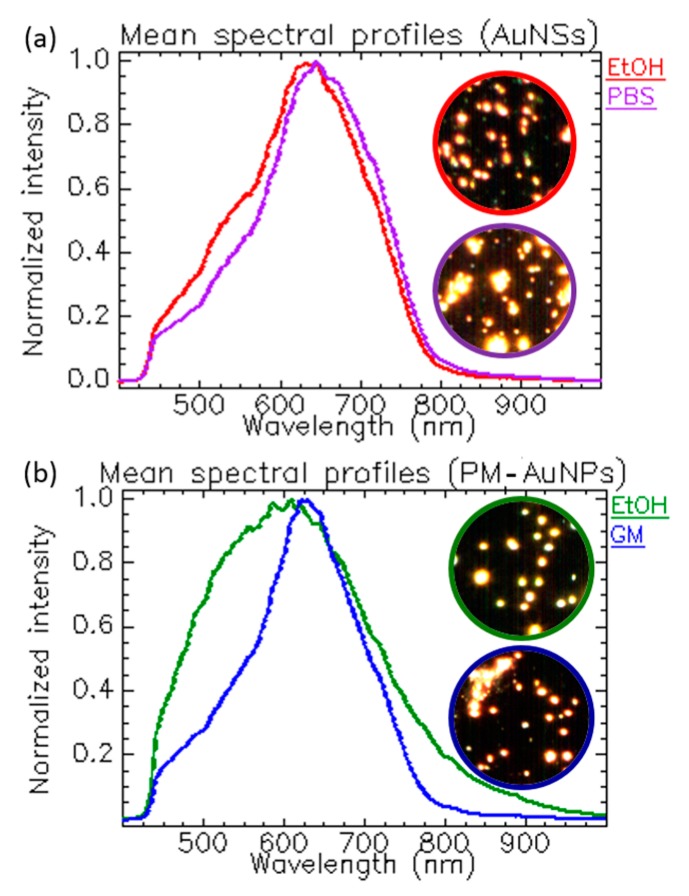
Influence of the solvents’ ionic strength and composition. (**a**) Spectral changes correlated with the agglomeration state of the AuNSs; (**b**) prominent spectral change in GM resulting from the formation of the so-called protein corona around the surface of the PM–AuNPs. AuNSs and PM–AuNPs were synthesized in our lab following synthesis methods published elsewhere [13,14]. CytoViva setup and ENVI 4.8 software were used for the hypercube acquisition and analysis.

All optical processes, including the scattering of light, are heavily influenced by the properties of the medium across the pathway of light. Biological environments are complex aqueous media rich in enzymes, proteins, sugars, peptides, electrolytes, among other molecules affecting the properties of the bulk in terms of ionic strength, viscosity, and refractive index. We wanted to study the impact of the composition of the dispersion medium on the NPs, so we carried out a spectral analysis of gold nanostars (AuNSs) in ethanol (EtOH) (a standard medium used for the storage of NPs) and phosphate-buffered saline (PBS) (a standard medium used in bio-related applications). The generated mean spectrum in ethanol showed a band located around 631 nm, while, in PBS, the band maximum slightly redshifted to 645 nm (Figure 5a and Figure S3). This correlated with an increase in size due to NP agglomeration caused by the high ionic strength of PBS that screened the charges. The insets corresponding to the dark field images of the NPs confirmed the agglomeration of the sample. The narrowing of the band could be associated with an increase in the sample homogeneity.

Next, we exposed plasmonic nanoparticles with different physicochemical properties compared to the nanostars (around 80 nm) to ethanol and cell growing medium (GM), a more complex medium with high ionic strength like the PBS and higher viscosity due to the presence of macromolecules. To this end, we used polymeric hollow nanocapsules whose cavity was covered with discrete gold nanoparticles (PM–AuNPs) (overall size 400 nm). Figure 5b shows the spectral profiles in both media. The complex content of the GM altered the viscosity, ionic strength, and refractive index of the bulk, generating a narrow and redshifted spectral band (Figure S4). The existing proteins affected the dielectric constant of the bulk and interacted with the NPs, forming a protein corona [12] which changed the size and the interfacial refractive index, ultimately resulting in different scattering profiles.

#### 1.3.4. Time Evolution of NPs Stored in Water or in a Biological Medium

**Figure 6:**
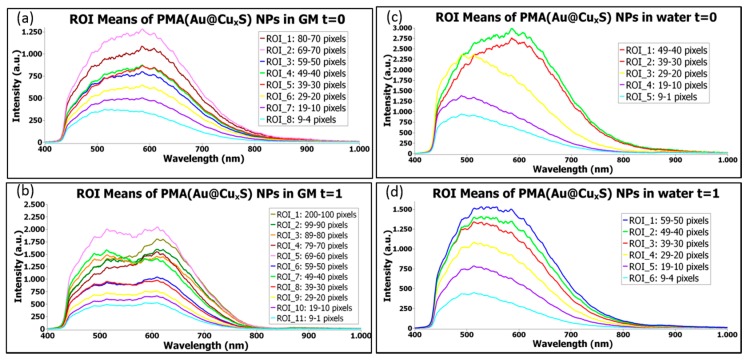
Temporal tracking AuCuS NPs spectral changes induced by the solvent’s viscosity. HEDFM tracked the scattering profiles of NPs dispersed in water (non-viscous medium) and cell GM (viscous medium) over time. The plots represent the mean spectral profiles of different regions of interest (ROIs) containing the spectra of particles with the same pixel size. The number of pixels associated with one particle forms its pixel size. We sorted the particle populations by pixel size at different time points, i.e., *t* = 0 and *t* = 1, corresponding to the time of immediate addition of the solvent and the time after 2–3 months of storage, respectively in GM (**a**) and (**b**); and water (**c**) and (**d**). In the SI (Figure S5), the evolution of the NPs after 8 months in GM is also shown [8].

Plasmonic nanoparticles are well characterized and present large scattering cross sections arising from their composition and morphology. They are the standards for HEDFM. In one of our recent studies, we investigated the time-induced morphological alterations in plasmonic AuCuS NPs [8]. Figure 6 presents the evolution of AuCuS NPs after 2–3 months in water and cell GM. From the wide mean spectral profiles of the NPs at *t* = 0 month (Figure 6a,c), we could conclude that the NPs per se scattered the light differently. This can be associated to the anisotropic geometry of the NPs derived from the non-concentric association of Au and CuS particles, two different materials with different scattering cross sections. No obvious variations were observed when the NPs were dispersed in water over time (Figure 6d). On the other hand, the spectral analysis of the NPs in GM, containing at least 10% of macromolecules like proteins, responsible for the formation of a protein corona around the NPs’ surface, clearly discriminated between two spectral-like subpopulations with time (Figure 6b). The shoulder in the 500-nm region probably arose from the redispersion of the NPs mediated by the protein corona. At long term (see in Figure S5 the SI of the NPs in GM after 8 months), there was a narrowing in the spectral profile that slightly shifted to the red. 

#### 1.3.5. Tracking NPs’ Evolution in the Intracellular Milieu 

**Figure 7:**
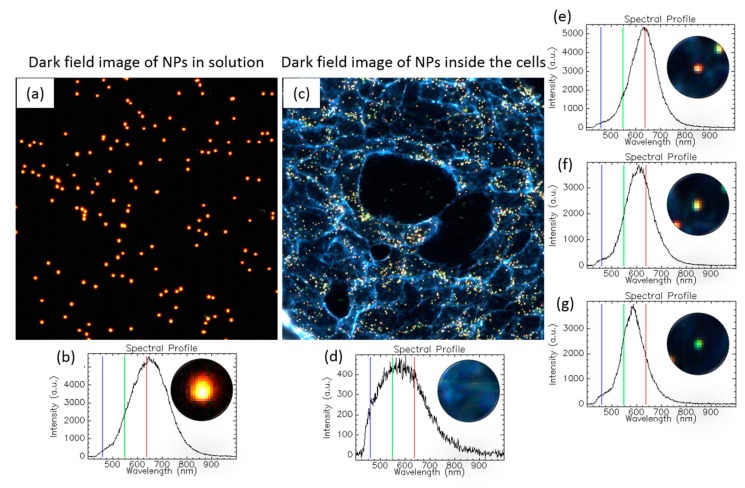
Spectral behavior of Au nanospheres (100 nm) inside and outside a cell. (**a**) Dark field image of the NPs in solution; (**b**) single spectral profile from one particle randomly selected; (**c**) dark field image of cells exposed to the NPs; (**d**) spectral signature of the cytoplasmic membrane; (**e**–**g**) particles showing different scattering behaviors in the intracellular milieu. All the plots show the RGB bars in which the dark field images are based. The hyperspectral images were acquired with the CytoViva setup and processed with ENVI 4.8 software.

HEDFM is an excellent tool for tracking spectral signatures in cell studies. The intracellular matrixes show a higher variation in chemical composition derived from the different cellular structures. Figure 7 shows the NPs behavior inside and outside cells (see detailed hyperspectral analysis of gold nanospheres alone and with cells in Figures S6, S7-A, and S7-B). The spectral profile of the cells compared to the AuNPs differs substantially, allowing optical and spectral differentiation. The spectral profile of the NPs in solution showed a band with a maximum located at 645 nm (Figure 7b), while the cell spectrum presented a wide band across the vis-NIR range (Figure 7d). The localization of the particles upon interaction with the cells was based on the different optical responses of the particles in the different compartments inside the cells. The latter can be translated in spectral shifts in the location of the band maxima at 633 nm (Figure 7e), 603 nm (Figure 7f), and 582 nm (Figure 7g), compared to the band maximum of the particles in solution at 645 nm. The absence of a red shift indicated that the NPs did not agglomerate inside the cells [12]. 

## 2. Biomedical Applications of HEDFM

Tracking the evolution of bio-nanomaterials is crucial to preserve their functionality [15,16,17]. Biological matrixes such as body fluids or tissues are considered more complex environments than the examples mentioned above. For instance, how NPs behave in the blood is key for nanomedicines designed for intravenous administration. Changes in the physicochemical properties of nanomaterials upon interaction with body fluids and tissues alter the pharmacokinetic response in terms of the drug biological behavior and potential toxicity [18]. In this section, we present examples of HEDFM applications in biomedicine.

**Figure 8:**
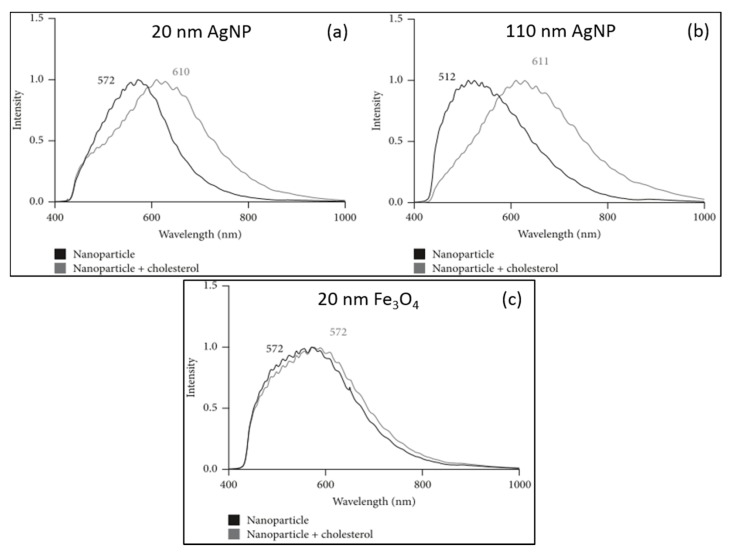
Impact of cholesterol on the optical properties of silver and iron oxide NPs (**a**) a 38 nm redshift is visible upon interaction of the 20 nm AgNP with cholesterol ; (**b**) a 99 nm redshift is visible, resulting from cholesterol interaction with the of 110 nm AgNP; (**c**) only spectral broadening can be observed for the 20 nm IONPs maintaining the spectral maximum at 572 nm [20].

Shannahan and co-workers studied the relation between nanoparticles and underlying diseases [19]. Atherosclerosis is associated with the capacity of macrophages to uptake cholesterol that leads to inflammation. Macrophages are also one of the first immune cells in charge of detecting and eliminating unidentified agents such as nanoparticles. Silver particles (AgNPs) and iron oxide particles (IONPs) may compete with cholesterol for the scavenger receptors that macrophages have on their surface, thus playing a key role in the metabolism and clearance of these different materials. To study the immune system interactions under cholesterol influence, the researchers used HEDFM to characterize the scattering properties of citrate-coated AgNPs of 20 and 110, and 20 nm polyvinylpyrrolidone (PVP)-coated IONPs, in the presence or absence of cholesterol (Figure 8). The interaction between cholesterol and the surface of NPs alters the hydrodynamic size, the ζ-potential, and the optical properties. Specifically, it was observed an important redshift of the spectral responses of both 20 and 110 nm AgNPs upon interaction with cholesterol. This shift could be correlated with changes in the refractive index of the nanoparticles resulting from cholesterol attachment. In the case of the PVP-coated IONPs, the changes were less obvious, while the location of the band maximum was preserved, and a spectral broadening occurred. It is worthy to say that both PVP and cholesterol but not citrate have similar refractive indexes. This explains the higher differences in the scattering properties for the citrate-coated nanoparticles compared to the PVP-coated counterparts of the same size. Concerning the NP–immune cell interactions, the study elucidated that macrophages could uptake almost all particles (AgNPs and IONPs) within the first 2 h of a 24 h incubation. Toxic events were not observed upon sequential or simultaneous exposure of macrophages to cholesterol and nanoparticles. The presence of cholesterol had no effect in the NP internalization pattern for the 20 nm particles (AgNPs and IONPs), nevertheless, it increased the uptake of the 110 nm AgNPs. Another example using AgNPs on mastocytes was also investigated by the group of Shannahan. They reported the degranulation of mastocytes, typically found in allergic reaction, investigating the effect of 20 nm AgNPs functionalized with different coatings (citrate or PVP). The activation of the mastocytes was not observed in the case of the 110 nm AgNPs for both coatings [18]. 

**Figure 9:**
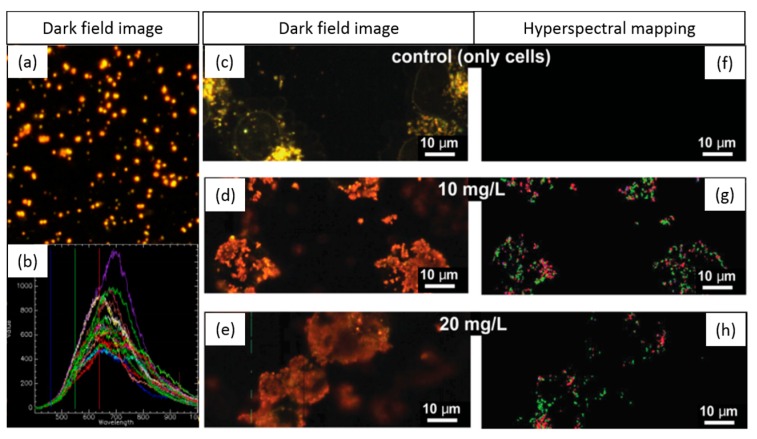
Mapping of hybrid nanostructures in human breast cancer cells to determine the optimal particle concentration for photothermal therapy. (**a**) Dark field image of the NPs; (**b**) spectral library endmembers; (**c**–**e**) dark field image of control cells and cells exposed to 10 and 20 mg/L NP concentrations; (**f**–**h**) hyperspectral imaging of the spectral library demonstrating the presence of the particles in the cells (false-colored image) [21].

**Figure 10:**
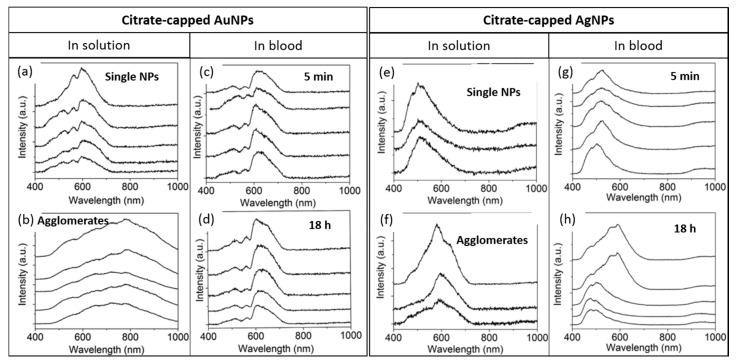
Determination of the agglomeration state of gold and silver NPs in blood. (**a**,**b**) AuNP spectral characterization at different agglomeration states; (**c**,**d**) spectral signatures of AuNPs in blood after 5 min and 18 h of exposure; (**e**,**f**) spectral responses of the AgNP upon agglomeration; (**g**,**h**) AgNPs in blood and their corresponding spectral profiles after 5 min and 18 h of exposure [13].

The ability to internalize hybrid plasmonic–superparamagnetic nanostructures by human breast cancer cells was reported in the work of Sotiriou et al [20]. A spectral library of the particles in water was generated and mapped in the different experimental conditions (Figure 9). Despite the heterogeneity of the spectral profiles, a great number of pixels were mapped in the case of cells exposed to nanoparticles, while for the control cells without particles no matches were found. The HEDFM method permitted the selection of the best concentration of nanoparticles from the two tested concentrations (10 and 20 mg L^−1^). The highest concentration of nanostructures was subsequently employed in the photothermal ablation assays, demonstrating their potential for cancer treatment. Jenkins and co-workers used HEDFM to examine the colloidal stability of nanoparticles in blood [21]. The study focused on gold and silver plasmonic nanomaterials, well-known for their unique optoelectronic properties and their widespread use in biological applications. Therefore, nanoparticles presenting different states of agglomeration, from single particles to macroscopic agglomerates, were exposed to blood. Next, the authors characterized the scattering responses and created the spectral libraries for each entity (Figure 10). Non-aggregated particles showed spectra with one wide main band, while the agglomerates presented complex spectral signatures with multiple contributions. Next, to evaluate the colloidal stability, single particles were exposed to blood and incubated for 18 h. The researchers recorded a data cube for *t* = 5 min and a data cube for *t* = 18 h of incubation. The generated spectral libraries concluded that the AuNPs presented higher colloidal stability over time. In the case of AgNPs, the researchers evidenced the presence of agglomerated particles in the blood, pointing out their potential toxicity for in vivo studies upon intravenous administration.

The investigation and analysis of nanomaterials in tissue sections using HEDFM require special attention. The steps required for the preparation of the samples are prone to change the scattering properties of the nanomaterial. For example, Sen et al. described a shift in the scattering spectra of gold-nanorods-labeled leucocytes (GNRLL) after exposing them to mounting media which presented a high refractive index [22]. They also reported that the hematoxylin–eosin staining (utilized in the identification of anatomical structures) interfered with the signal of GNRLL in spleen and retina tissues. Moreover, they had to preserve the paraffin layer to avoid the loss of GNRLL present inside the blood vessels (non-anchored). Thus, while the optical recognition of the GNRLL in the dark field image was possible, the spectral characterization was disabled because of the inherent procedures during tissue processing. 

Wen et al. were able to spectrally differentiate the presence of three different nanosystems inside brain histological sections [17,23]. They contemplated the following nanoparticles: theranostic liposomes containing apomorphine and quantum dots (QDs), bare liposomes, and free QDs. Firstly, they spectrally characterized the different NPs evidencing that each of them presented a specific spectral signature. Although all the particles exhibited the ability to scatter light from the visible to the near-infrared region, the shapes of the spectral profiles were different from each other and, thus, distinguishable on tissue samples. All the particles were detected except the free QDs during the mapping procedure of the spectral libraries in the brain data cubes. Despite the fact that both types of liposomes were found, the multifunctional liposomes were detected at a significantly higher concentration than the bare liposomes, validating their brain-targeting functionality.

## 3. Conclusions

Nanomedicine is an emerging discipline capable to offer new perspectives to current medical limitations. Novel imaging technologies are necessary tools to improve the fundamental knowledge in bio-nano-interactions promoting advances at the diagnostic and therapeutic levels. In this context, the HEDFM presents straightforward and rapid analysis of nanomaterials in their colloidal dispersion state. The latter is of great importance to better understand the biointeractions of nanomaterials and study their fate in the native aqueous environment of any biological system. However, the huge amount of the generated data forming the hypercube require copious processing and careful analysis to extract appropriate conclusive information. Many research reports use HEDFM to present spectral differences or similarities, lacking both a linkage with the physicochemical background and a profound interpretation of the findings. Nanomaterials with well-defined optical properties are, by far, better candidates for reproducible and representative analyses minimizing the variations derived from particle polydispersity. As a final remark, we anticipate that the HEDFM technology will be part of the growing toolbox for the bionanotechnological development in modern health sciences.

This review highlights the great potential of HEDFM technology to shed light on the dynamic interplay between nanomaterials and biological systems. Nanocomposites, at the single particle level or collectively, can be tracked by exploiting the dark field optics and characterized by their spectral signatures. Furthermore, with this technique, a non-invasive and label-free bioanalysis is possible, spanning from live cell and tissue studies to in vivo imaging of small organisms. We engaged HEDFM analysis to elucidate the relationship between nanomaterials and their environment through the monitoring of the optical response. We considered intrinsic factors of the nanomaterials, such as morphology and composition, as well as extrinsic stimuli related to their environment and to time. Examples of nanomaterial interactions in cells, blood, and tissues were presented for their biomedical relevance, and to show the applicability of the methodology in complex biological matrixes. To facilitate a comprehensive understanding of the methodology, we introduced the reader to the hyperspectral data acquisition and analysis.

## Supplementary Materials

The following are available online at http://www.mdpi.com/1996-1944/11/2/243/s1, Figure S1: Mean spectral responses and dark field images of PS, PS-RB, PS-QD, and PS-MB particles. Figure S2: Hyperspectral analysis of AuCuS NPs at pH 4 and 7. Figure S3: Hyperspectral analysis of AuNSs in ethanol and PBS. Figure S4: Hyperspectral analysis of PM-AuNPs in ethanol and GM. Figure S5: Spectral response of AuCuS NPs in GM after 8 months. Figure S6: Mean and single particle spectral analysis of 100 nm AuNPs in water. Figure S7-A: Spectral behavior of 100 nm AuNPs inside the cell. Figure S7-B: Cell maintenance and sample preparation.
