# Supplementary material for: Hyperspectral-Enhanced Dark Field Microscopy for Single and Collective Nanoparticle Characterization in Biological Environments
Source: Materials (Basel). 2018 Feb 6;11(2):243. doi: 10.3390/ma11020243 (PMC5848940; doi:10.3390/ma11020243)
Supplement: Supplementary file 1 [file materials-11-00243-s001.pdf]

Supplementary information

Hyperspectral enhanced dark field microscopy for single and collective nanoparticle characterization in biological environments

Paula Zamora-Perez [1, #], Dionysia Tsoutsi [1, #, *], Ruixue Xu [1] and Pilar Rivera_Gil [1,*]

[1] Integrative Biomedical Materials and Nanomedicine Lab, Department of Experimental and Health Sciences (DCEXS), Pompeu Fabra University (UPF), PRBB, Barcelona, Spain;

[#] Both authors contributed equally to the manuscript

* Correspondence: dionysia.tsoutsi@upf.edu , pilar.rivera@upf.edu ; Tel.: +34 933160918

Figure S1

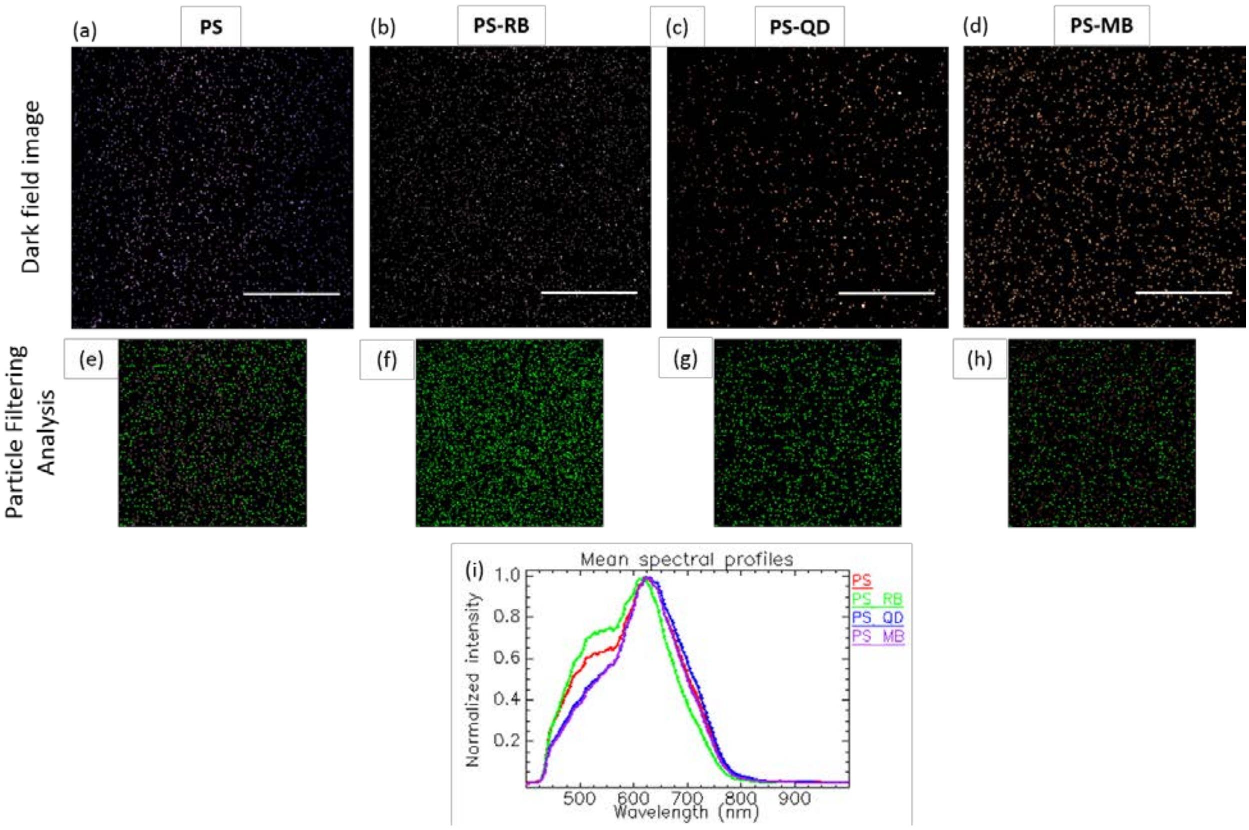

Figure S1: Mean spectral responses of polystyrene (PS) beads alone and loaded with different cargos (a-d) dark field images of the bare PS, rhodamine-B-loaded PS (PS-RB), quantum-dot-loaded PS (PS-QD) and methylene-blue-loaded PS (PS-MB) particles. The particles are well dispersed as can be seen from the images; (e-h) false-colored dark field images of each sample resulting from the particle filtering analysis; (i) the mean spectral profiles for each sample calculated from the green false-colored pixels as shown above (e-h). In specific, we have considered 831 particles (17418 pixels) for PS, 2088 particles (25306 pixels) for PS-RB, 782 particles (12288 pixels) for PS-QD, and finally, 633 particles (13791 pixels) for PS-MB. The high number of particles considered for the calculation of the corresponding mean spectra, demonstrates the statistical relevance of the analysis. The scale bar corresponds to 50 μm.

Figure S2

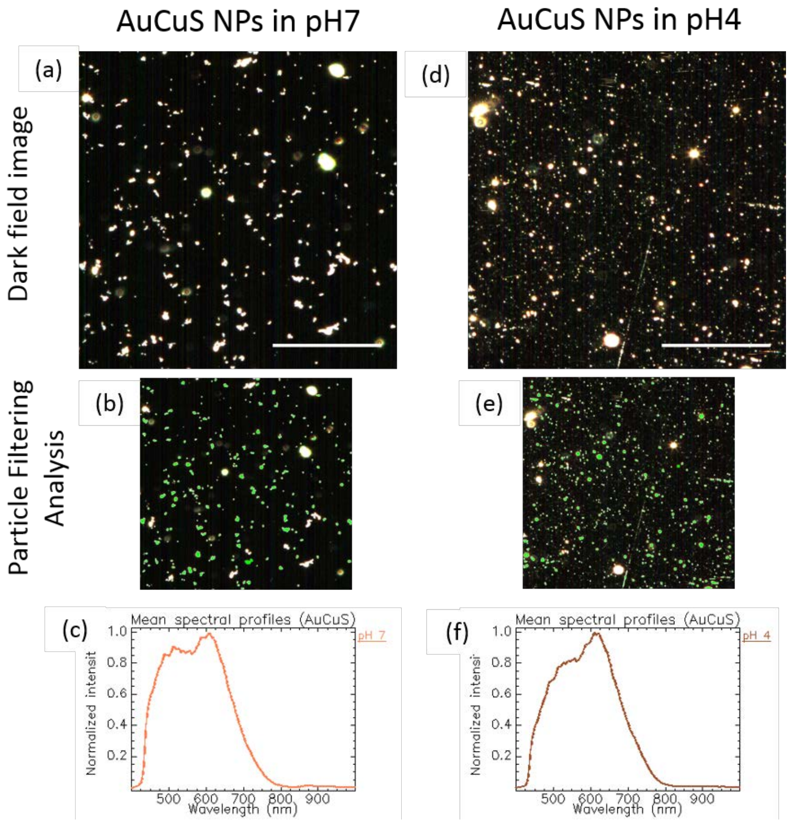

Figure S2: Hyperspectral analysis of AuCuS NPs at acidic and neutral pH mimicking the intravesicular and cytosolic/extracellular cell areas, respectively. (a-c) False-colored dark field images showing 242 AuCuS NPs in green and the generated mean spectrum at pH 7. (d-f) False-colored dark field images showing 588 particles in green and the generated mean spectrum at pH 4. (scale bar: 50 μm)

Figure S3

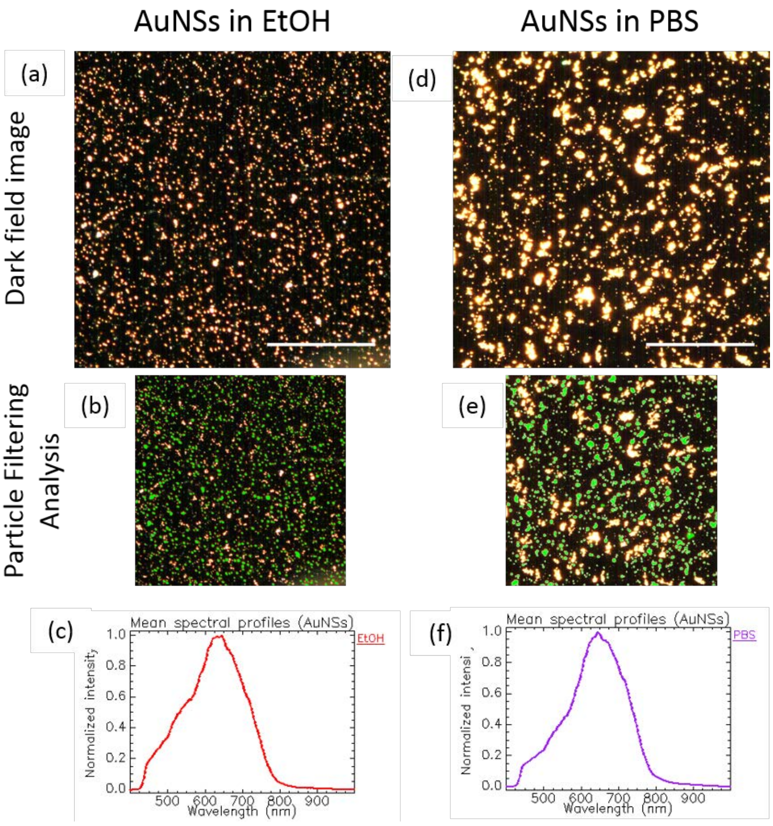

Figure S3: Hyperspectral analysis of AuNSs exposed to different solvent and ionic strength. (a-c) Green false-colored dark field images containing 987 NPs, and the calculated mean spectrum in ethanol; (d-f) dark field images containing 882 NPs, and the calculated mean spectrum in PBS. (scale bar: 50 µm)

Figure S4

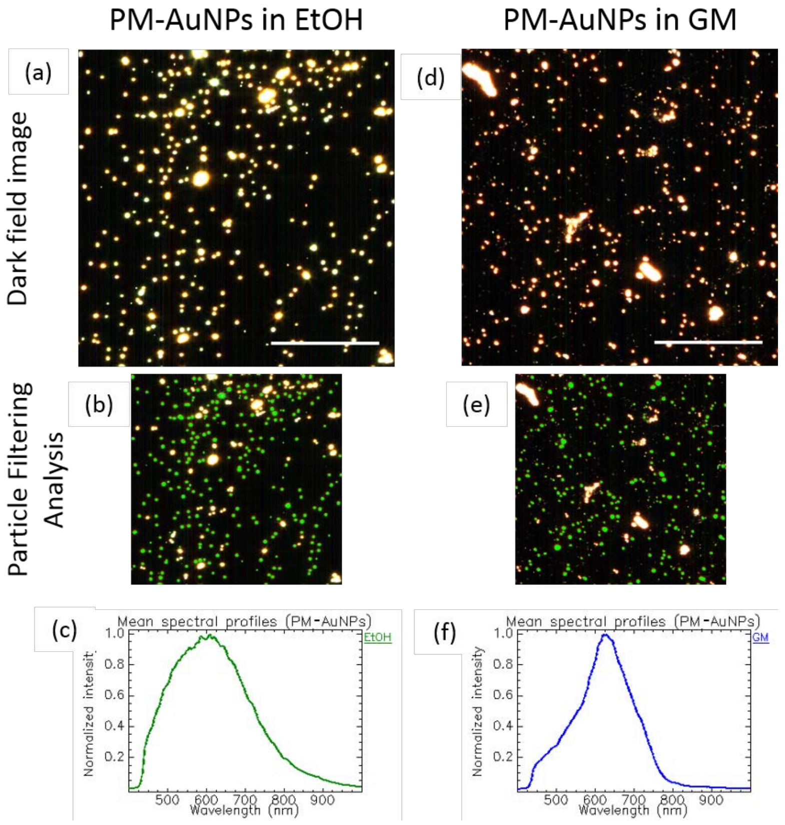

Figure S4: Hyperspectral analysis of the PM-AuNPs varying the solvents´ nature and complexity. (a-c) Green false-colored dark field images containing 253 NPs, and the calculated mean spectrum in ethanol; (d-f) dark field images containing 254 NPs, and the calculated mean spectrum in GM. (scale bar: 50 µm)

Figure S5

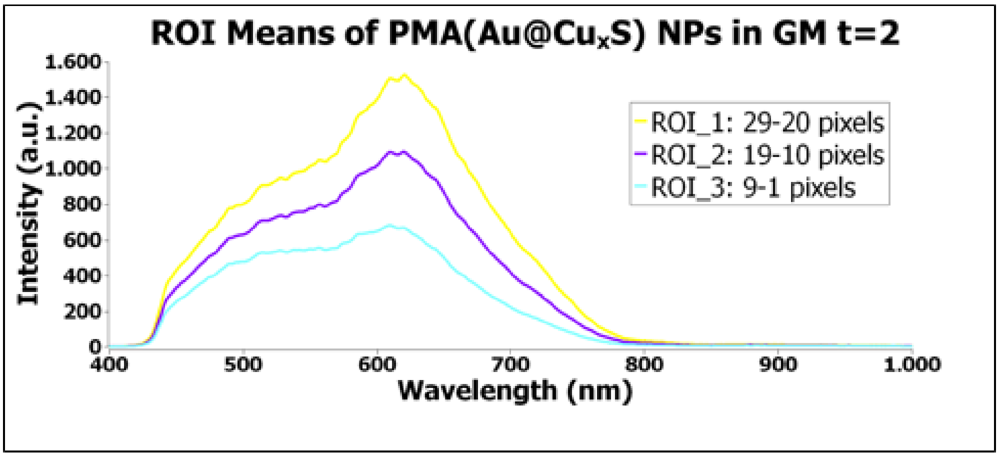

Figure S5: Spectral response of gold-copper sulfide NPs (AuCuS NPs) in cell growth media (GM) over time. The mean spectral profiles of 3 different regions of interest (ROI) of AuCuS NPs sorted by the number of pixels represented in the data cube are provided. In specific, the spectral information was obtained after 8 months of exposure of AuCuS NPs to GM.

Figure S6

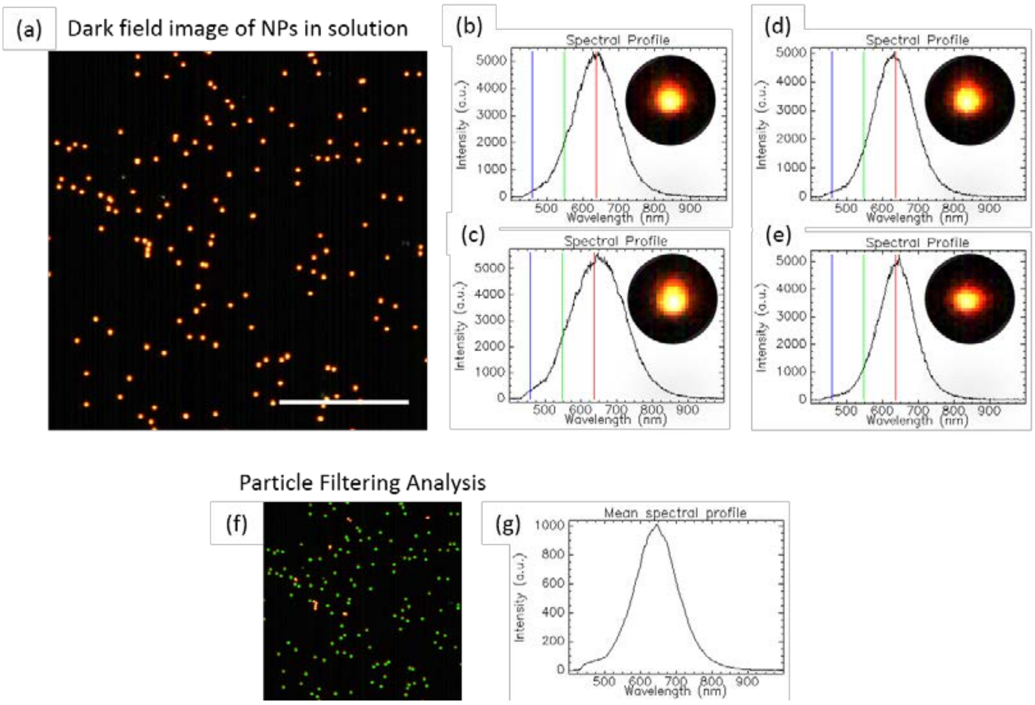

Figure S6: Mean and single particle hyperspectral analysis of 100 nm gold nanospheres in water. (a) Dark field image of the NPs in water; (b-e) single spectra at the maximum scattered light intensity coming from individual particles randomly selected. The four spectral profiles show similar band maximum wavelengths: 645 nm, 645 nm, 630 nm, and 645 nm corresponding to b, c, d, and e plots, respectively; (f) false-colored dark field images image showing 136 particles in green; (g) mean spectral profile with band maximum at 645 nm. Scale bar: 50 μm.

Figure S7 - A

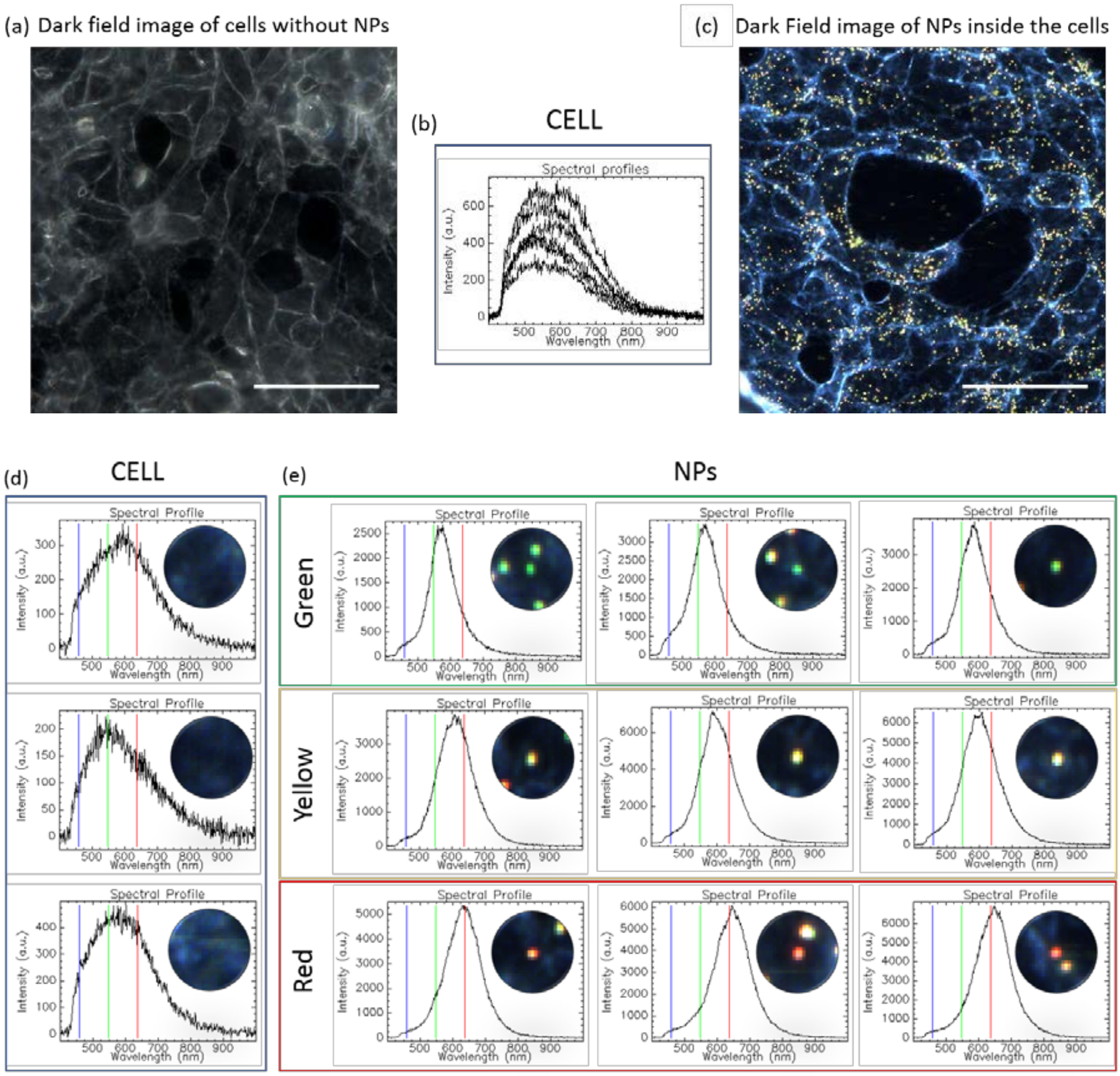

Figure S7: Spectral behavior analysis of gold nanospheres (100 nm) inside the cell. (a) Dark field image of HEK 293T human embryonic kidney cells non-exposed to nanoparticles; (b) Spectral profiles from different cell areas randomly selected; (c) Dark field image of HEF 293T cells exposed to AuNPs after 24-hour incubation; (d) spectral profiles coming from different cell areas indicating spectral similarity with the control cells; (e) spectral profiles of AuNPs categorized upon their different scattering responses in the green, yellow and red regions of the visible window. The band maxima in each color category are: 573 nm, 571 nm, and 582 nm for the green particles, 603 nm, 588 nm, and 588 nm for the yellow, and finally, 633 nm, 640 nm, and 645 nm for the red. Again, different intracellular conditions provoke changes at the optical responses of the nanomaterials. All the plots show the red-green-blue (RGB) bars in which the dark field images are based. (Scale bar: 50 μm).

Figure S7 - B

Cell maintenance and sample preparation of figure S7: The HEK293T cell line was provided by the laboratory of molecular physiology, department of experimental and health sciences from Pompeu Fabra's University. The cells were cultured following ATCC recommendations using: Dulbecco's modified Eagle's medium (DMEM) with high glucose content and sodium pyruvate (110 mg/L, n (Gibco, ref. 41966029) supplemented with 10% fetal bovine serum (Hyclone, ref. SH30088.02), 200 mM L-glutamine (Hyclone, ref. SH30034.01), and 100 U/mL penicillin with 100 μg/mL streptomycin (Hyclone, ref. SV30010). The passaging of cells was performed twice per week when reaching 80% confluency, using 0.05% trypsin-EDTA (Gibco, ref. 25300062) and Hank's balanced salt solution (HBSS) (Cultek, ref. BE10-547F).

HEK293T cells were seeded on coverslips using a 6-well plate and placed overnight in the incubator in an atmosphere of humidified air with 5% CO_2 at 37ºC. 5.6x10^7 citrate-coated gold nanospheres of 100 nm were added to cells at 75% confluency. After 24 hours of incubation the coverslips were placed into clean microscope slides and acquisitions were obtained for each sample. Control cells without nanoparticles were imaged as well.
